# The complete mitochondrial genome of *Sardinella zunasi* (Clupeiformes: Clupeidae)

**DOI:** 10.1080/23802359.2021.1902407

**Published:** 2021-03-24

**Authors:** Moo-Sang Kim, Jinho Kim, Tae-Wook Kang, Uiseok Jeong, Kang-Rae Kim, In-Chul Bang

**Affiliations:** a The MOAGEN, Daejeon, Republic of Korea; b Department of Life Science & Biotechnology, Soonchunhyang University, Asan, Republic of Korea

**Keywords:** Complete mitochondrial genome, Clupeoidei, *Sardinella zunasi*, phylogenetic analysis

## Abstract

The complete mitogenome of *Sardinella zunasi* was determined by next-generation sequencing. The *S*. *zunasi* mitogenome was a circular 16,307 bp molecule that contained 13 protein-coding genes, 22 tRNA genes, 2 rRNA genes, and one control region (D-loop). The gene arrangement was consistent with other *Sardinella* mitogenomes. The phylogenetic relationships of 29 Clupeoidei species based on 13 protein-coding genes from the available mitogenomes were analyzed. *Sardinella zunasi* clustered with *Sardinella* among Clupeidae, suggesting a closer relationship with this genus. These results will be useful for understanding the phylogenetic relationships, taxonomic classification, and phylogeography of the genus *Sardinella* relative to other genera of Clupeoidei.

*Sardinella zunasi* (Japanese sardinella) is a ray-finned fish in the family Clupeidae, which includes herrings and sardines. It is native to the northwestern Pacific, where it occurs near shore along the Asian coastline from southwestern Korea and southern Japan to Taiwan. It is commercially important in Korea, Japan, and China (Wang et al. 2008) and has been heavily fished and is considered overexploited. Numerous ecological and morphological studies have examined the species; however, the complete mitochondrial DNA (mtDNA) sequence of this species has not been reported (Kim et al. 2001).

A specimen was bought at a Korean fish market and stored at Soonchunhyang University, Republic of Korea, under accession no. SUC-30000. The species identification of the specimen was confirmed morphologically.

Total genomic DNA was extracted from muscle tissue using a NucleoSpin Tissue Kit (Macherey-Nagel, Düren, Germany) and a genomic library for next-generation sequencing (NGS) was constructed using the MGIEasy DNA Library Prep Kit (MGI Tech, Shenzhen, China). After obtaining raw NGS data on an MGISEQ-2000 (MGI Tech), the mtDNA sequence of a circular 16,307 bp molecule was assembled using CLC Assembly Cell 5.1.1 (Qiagen, Hilden, Germany), and the assembled sequence was annotated using the web-based automatic MITOS annotation server (Bernt et al. 2013).

An NCBI BlastN (Johnson et al. 2008) search of the complete mtDNA sequence showed 98.37% identity to sequence NC_039553 of the *Sardinella lemuru* mitochondrion (Jiang et al. 2017). Therefore, the full mitochondrial sequence of *S*. *zunasi* has not been registered in the GenBank database, and this sequence is presumed to have high similarity with the mitochondrial sequence of *S*. *lemuru*. We performed a detailed NCBI BlastN search using the sequences of the COX1, 12S rRNA, and 16S rRNA regions of *S*. *zunasi*, which showed sequence identities of 99.64–99.69%, 99.42–100%, and 99.64–100%, respectively, with reported *S*. *zunasi* sequences and 99.54%, 99.90%, and 94.45% with *S*. *lemuru* sequences. The comparison of these regions confirmed that our mtDNA sequence was more similar to *S*. *zunasi* than to *S*. *lemuru*. Therefore, the mtDNA sequence completed here was for *S*. *zunasi*. This sequence was registered in GenBank under accession no. MW118114.

The complete mitogenome of *S*. *zunasi* (MW118114) was 16,307 bp in length and consisted of 13 protein-coding genes (PCGs), 22 tRNAs, two rRNAs (12S and 16S), and a non-coding region including the putative control region (D-loop). The *S*. *zunasi* gene arrangement was identical to those of its relatives: *S*. *lemuru* (Jiang et al. 2017), *Sardinella melanura* (Andriyono et al. 2019), *Sardinella fijiensis* (Wang et al. 2019), *Sardinella longiceps*, and *Sardinella gibbosa* (Sebastian et al. 2017). Twelve PCGs were on the heavy strand, while ND6 was on the light strand. All of the tRNAs were predicted to form typical stem-loop secondary structures, as in *S*. *lemuru*. The noncoding D-loop region was located between tRNA-Pro and tRNA-Phe. All PCGs had the typical ATG start codon, except for COX1 (ATC), COX2 (GTG), and ND6 (GTG). Incomplete stop codons were identified in ND2, COX2, COX3, ND3, ND4, and CYTB, as in S. *melanura*.

A maximum-likelihood phylogenetic tree of *S*. *zunasi* and 28 other Clupeoidei species was constructed based on the 13 protein-coding genes using MEGA-X (Kumar et al. 2018). Interestingly, this showed that *S*. *zunasi* clustered with *S*. *lemuru*, suggesting a very close relationship, confirming that the current sequence belongs to *Sardinella* (Figure 1). To confirm that the species identification of *S*. *lemuru* NC_039553 was correct, a p-distance analysis using the complete mitogenome sequences of *Sardinella* species gave values of 0.196–0.214 with other species in *Sardinella*, while the distance between *S*. *zunasi* and *S*. *lemuru* was 0.008. In a more detailed analysis, the p-distances were determined for the other *cox1* genes of *S*. *zunasi* and *S*. *lemuru* in GenBank and ranged from 0.0031 to 0.0053 between the *cox1* genes of *S. zunasi*; NC_039553 *S*. *lemuru* had a value of 0.0044, while other *S*. *lemuru* sequences had values of 0.1799–0.1928 and *Sardinella maderensis* had a value of 0.1920. Therefore, NC_039553 was verified to be the *S*. *zunasi* mitochondrial sequence. The complete mtDNA sequence of *S*. *zunasi* should improve the taxonomic classification of *Sardinella* species with similar mtDNA sequences.

**Figure 1.**
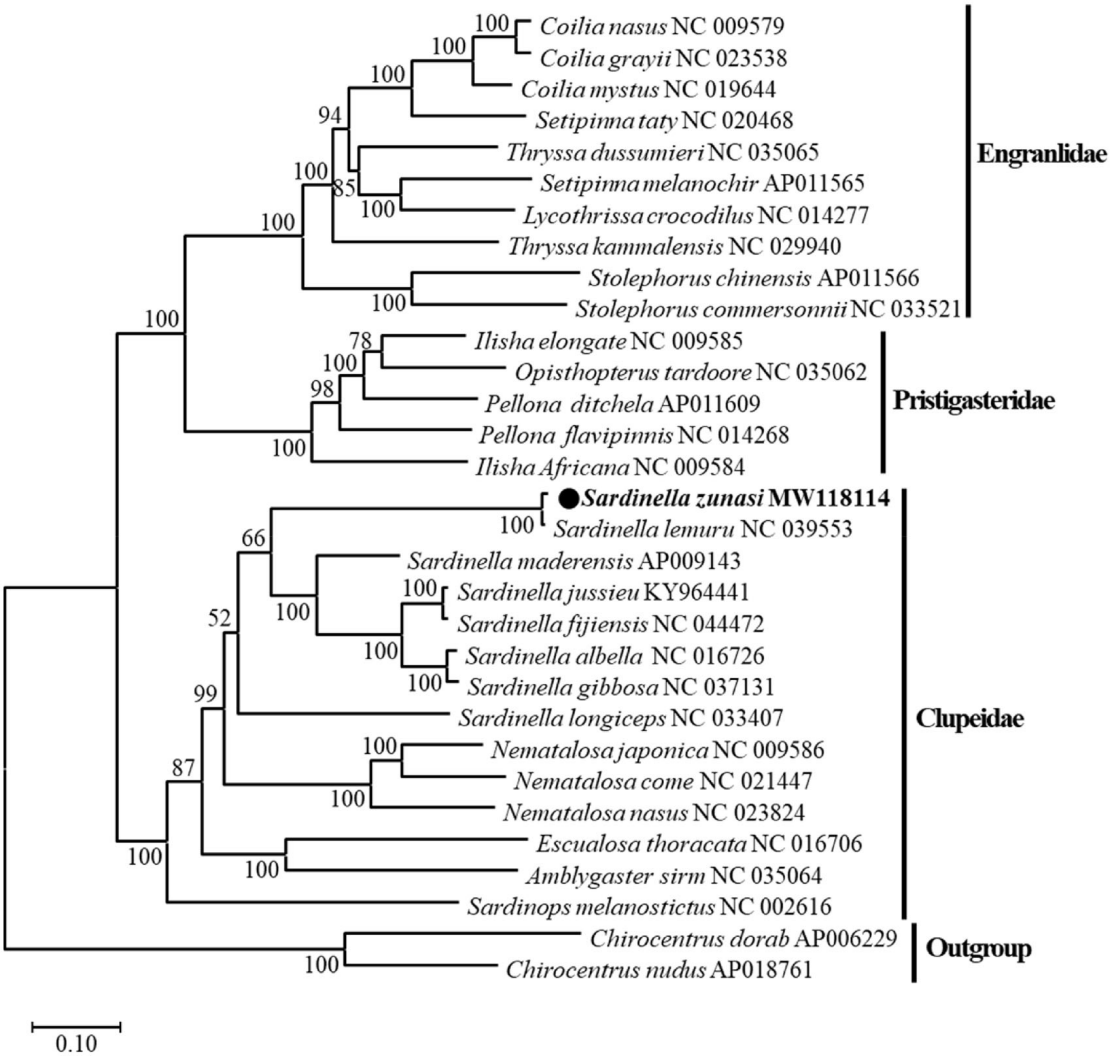
A maximum-likelihood phylogenetic tree of Clupeoidei based on 13 protein-coding genes from the complete mitogenomes was constructed in MEGA-X with the GTR + Gamma + I substitution model. The bootstrap values are based on 1000 replications. The number at each node is the bootstrap probability. The number after the species name is the GenBank accession number. The black circle indicates the species studies here.

## Data Availability

The genome sequence data that support the findings of this study are openly available in GenBank of NCBI at (https://www.ncbi.nlm.nih.gov/) under the accession no. MW118114. The associated BioProject, SRA, and Bio-Sample numbers are PRJNA695411, SRX9967975 and SAMN17613384, respectively.
